# *Apostasia* Mitochondrial Genome Analysis and Monocot Mitochondria Phylogenomics

**DOI:** 10.3390/ijms24097837

**Published:** 2023-04-25

**Authors:** Shi-Jie Ke, Ding-Kun Liu, Xiong-De Tu, Xin He, Meng-Meng Zhang, Meng-Jia Zhu, Di-Yang Zhang, Cui-Li Zhang, Si-Ren Lan, Zhong-Jian Liu

**Affiliations:** 1 College of Forestry, Fujian Agriculture and Forestry University, Fuzhou 350002, China; 3210422027@fafu.edu.cn (S.-J.K.); fjliudk@163.com (D.-K.L.); tttxd163@163.com (X.-D.T.); 5220422102@fafu.edu.cn (X.H.); 1220428020@fafu.edu.cn (M.-M.Z.); 1200455008@fafu.edu.cn (M.-J.Z.); 2 Key Laboratory of National Forestry and Grassland Administration for Orchid Conservation and Utilization at College of Landscape Architecture and Art, Fujian Agriculture and Forestry University, Fuzhou 350002, China; diyangzhang@126.com (D.-Y.Z.); cuilizhang@fafu.edu.cn (C.-L.Z.)

**Keywords:** *Apostasia*, mitochondrial genome, phylogenetic analysis, monocots

## Abstract

*Apostasia shenzhenica* belongs to the subfamily Apostasioideae and is a primitive group located at the base of the Orchidaceae phylogenetic tree. However, the *A. shenzhenica* mitochondrial genome (mitogenome) is still unexplored, and the phylogenetic relationships between monocots mitogenomes remain unexplored. In this study, we discussed the genetic diversity of *A. shenzhenica* and the phylogenetic relationships within its monocotyledon mitogenome. We sequenced and assembled the complete mitogenome of *A. shenzhenica*, resulting in a circular mitochondrial draft of 672,872 bp, with an average read coverage of 122× and a GC content of 44.4%. *A. shenzhenica* mitogenome contained 36 protein-coding genes, 16 tRNAs, two rRNAs, and two copies of *nad4L*. Repeat sequence analysis revealed a large number of medium and small repeats, accounting for 1.28% of the mitogenome sequence. Selection pressure analysis indicated high mitogenome conservation in related species. RNA editing identified 416 sites in the protein-coding region. Furthermore, we found 44 chloroplast genomic DNA fragments that were transferred from the chloroplast to the mitogenome of *A. shenzhenica*, with five plastid-derived genes remaining intact in the mitogenome. Finally, the phylogenetic analysis of the mitogenomes from *A. shenzhenica* and 28 other monocots showed that the evolution and classification of most monocots were well determined. These findings enrich the genetic resources of orchids and provide valuable information on the taxonomic classification and molecular evolution of monocots.

## 1. Introduction

Mitochondria are organelles that play a crucial role in plant productivity and development by being responsible for energy conversion, biosynthesis, and signal transduction [1]. The plant mitogenome is complex and diverse in terms of size, structure, number of repeats, and coding genes [2]. In addition, it has extensive horizontal gene transfer (HGT) and RNA-editing mechanisms that ensure mitochondrial function and stable gene expression [3,4]. Most seed plants inherit chloroplasts (cp) and mitochondria (mt) from their maternal parent. This genetic mechanism reduces the influence of paternal lines, making it easier to study genetics [5,6]. Thus, cp genomes and mitogenomes have been extensively analysed to understand taxon classification and evolution [7]. However, compared to plastid genomes, mitogenomes with lower evolutionary rates are more suitable for studying the phylogenetic relationships of families, orders, or higher taxonomic elements.

Monocots are one of the most diverse, ecologically important, and economically important terrestrial plant lineages with approximately 77 families and 85,000 species, accounting for 21% of angiosperms [8,9]. Despite their remarkable species diversity, only 30 monocots, including three orchids (*Gastrodia elata*, *Platanthera zijinensis*, *P. guangdongensis*) [10,11] mitogenomes have been published in the NCBI GenBank database due to the complexity of mitogenome structure and sequence, which limited the study and utilization of these excellent crops. At present, the phylogenetic relationships of monocots are mainly studied in cp genomes and mitochondrial gene segments, and the phylogenetic relationship of mitogenome is still unclear [12,13]. Therefore, it is extremely important to provide further evidence for the systematic relationship between monocot lineages using mitogenome.

Orchidaceae is the largest family of angiosperms and monocots with over 700 genera and approximately 28,000 species [14]. *Apostasia* is one of the two genera that form the subfamily Apostasioideae, which is a primitive group located at the base of the Orchidaceae phylogenetic tree [15]. Moreover, *Apostasia* has special morphology that demonstrates “primitive” traits similar to those of *Curculigo crassifolia*, are considered the closest to the “pseudoorchids” speculated by Darwin. This makes them ideal for studying orchids and phylogenetic evolution [16]. Therefore, we conducted a mitogenome study of *A. shenzhenica* to provide genetic resources for further studies on the evolution of Orchidaceae. Meanwhile, we combine the available mitogenomes of monocots to provide further evidence for the phylogenetic relationships of monocots.

In this study, we used second-generation sequencing techniques to de novo assemble the mitogenome of *A. shenzhenica* and systematically analysed gene content, repetitive sequences, selective pressure, and RNA editing sites. We investigated the gene transfer between the chloroplast and mitogenomes of *A. shenzhenica*. In addition, we explored the phylogenetic relationships among *A. shenzhenica* and 28 monocot species using the mitogenome, which provided valuable information on the taxonomic classification, molecular evolution, and breeding of monocots.

## 2. Results

### 2.1. Mitogenome Structure and Gene Content

**Figure 1:**
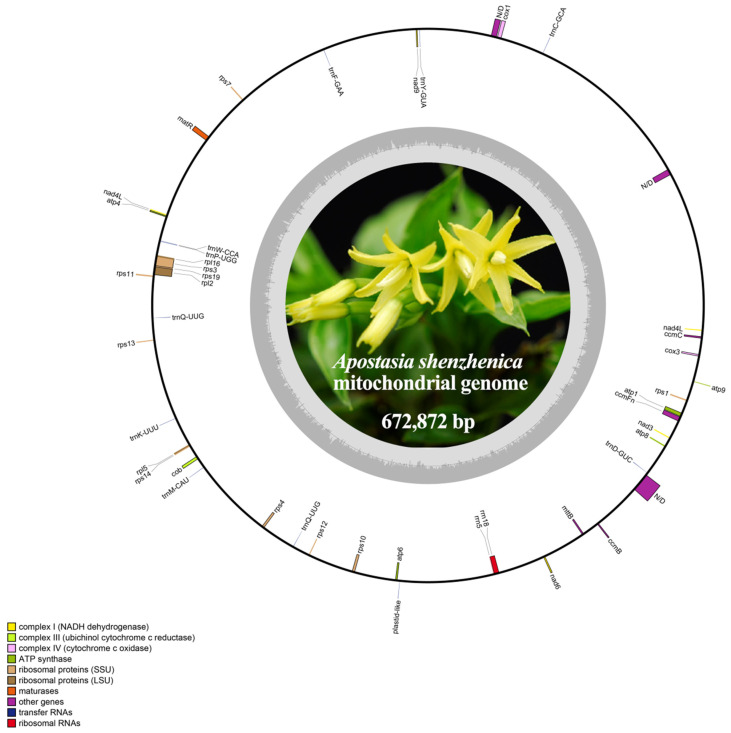
Map of the mitogenome of *A. shenzhenica*. The genes inside and outside the circle are transcribed in the clockwise and counterclockwise directions, respectively. Genes belonging to different functional groups are shown in different colors. The innermost darker gray corresponds to GC-content, while the lighter gray corresponds to AT content.

The genome sequence of *A. shenzhenica* was uploaded to GenBank (accession number: OQ645347). We assembled a 672,872 bp length mitogenome of *A. shenzhenica* using 18 Gb Illumina sequencing data and manually displayed a circular structure (Figure 1). The nucleotide composition of the mitogenome was 27.8% A, 27.8% T, 22.3% G, 22.1% C, and 44.4% GC. In addition, we identified 54 mitochondrial genes in the *A. shenzhenica* mitogenome, including 36 protein-coding genes, two rRNA genes, and 16 tRNA genes (Table 1 and Table S1). Protein-coding genes (PCGs) accounted for 4.21% of the total mitogenome, whereas tRNA and rRNA genes accounted for only 0.17% and 0.31%, respectively. The rest of the mitogenome contained noncoding sequences, such as introns, intergenic spacers, and potential pseudogenes. There were 57 repeat pairs with a length of >50 bp, occupying 1.38% (9298 bp) of the mitogenome. Interestingly, two copies of *nad4L* genes were detected.

The depth of coverage across the entire mitogenome was relatively even, indicating the continuity of our assembly (Figure 1). Overlaps and intervals exist between adjacent genes in the mitogenome. We identified three overlapping sequences. Overlapping sequences were located between *rp15* and *rps14*, *rp116* and *rps3*, and *rps19* and *rps3*. A total of 50 gene spacer regions with lengths ranging from 4 to 67,130 bp were identified. The largest spacer region was between *nad5* and *trnC-GCA*, with a length of 67,130 bp. Furthermore, most PCGs of *A. shenzhenica* have no introns; six of the annotated genes contained introns, five of them (*nad5*, *ccmFc*, *rpl2*, *rps3* and *rps10*) included an intron and *nad7* had four (Table 1). Analysis of the whole mitogenome sequence has putative group II intron segments near each exon. Then, we set the minimum size to 200 bp in Geneious and discovered that these genes have one or more ORFs with unknown function, laying in the middle region between its exons. For example, *ccmFc* intron has two ORFs with complete start and stop codons.

Except for *mttB*, which used TTG, most protein-coding genes used ATG as the start codon. In several higher plant mitogenomes, the start codons of the *mttB* gene were unclear [17,18]. The stop codons used in *A. shenzhenica* mitochondrial PCGs were TAA (19 genes; *atp4*, *atp6*, *atp8*, *atp9*, *ccmFc*, *ccmFn*, *cox1*, *mttB*, *nad3*, *nad4L*, *nad6*, *nad9*, *rpl5*, *rpl16*, *rps1*, *rps4*, *rps7*, *rps10*, and *rps11*), TAG (6 genes; *cob*, *matR*, *nad7*, *rpl2*, *rps3*, and *rps14*), and TGA (9 genes; *atp1*, *ccmB*, *ccmC*, *cox3*, *nad5*, *rps12*, *rps13*, *rps19*, and *psaJ*).

### 2.2. Codon Usage Analysis of PCGs

**Figure 2:**
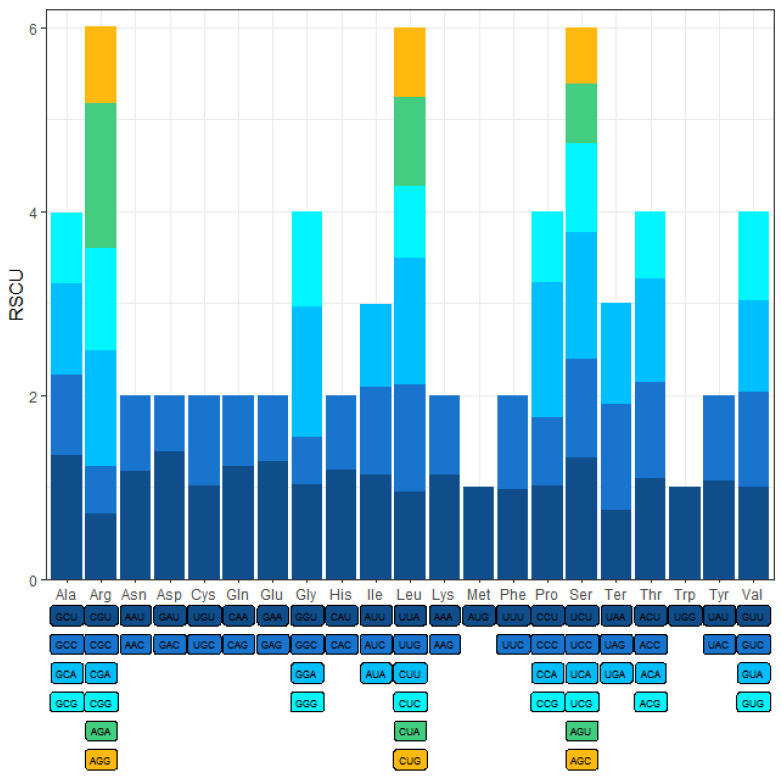
Relative synonymous codon usage (RSCU) in *A. shenzhenica* mitogenome. Codon families are shown on the x-axis. RSCU values are the number of times a particular codon is observed relative to the number of times that codon would be expected for a uniform synonymous codon usage.

To ATG was the most frequent start codon for 36 protein-coding genes (Table 1); the *mttB* gene was an exception with the initiating codon TTG. Three stop codons (TAA, TAG, and TGA) were identified. These results indicated that RNA editing from C to U does not occur at the start or stop codon. The relative synonymous codon usage (RSCU) values of *A. shenzhenica* are displayed in Figure 2. The results showed that the 36 protein-coding gene regions had 9379 codons, excluding termination codons (Table 2). The most frequently used codons were UUC and UUU (for Phenylalanine; Phe) and CUU (for leucine; Leu), whereas CGU and CGC (for Serine; Ser) and GGC (for tryptophan; Trp) were rarely found. This may explain the negative base skew (AT, GC) of the PCGs.

### 2.3. Substitution Rates of PCGs

The non-synonymous-to-synonymous substitution ratio (Ka/Ks) is important in genetics for assessing the magnitude and direction of natural selection acting on homologous genes among divergent species. It is commonly accepted that Ka/Ks indicates neutral evolution when it equals one, positive selection when it is greater than one, and negative selection when it is less than one.

**Figure 3:**
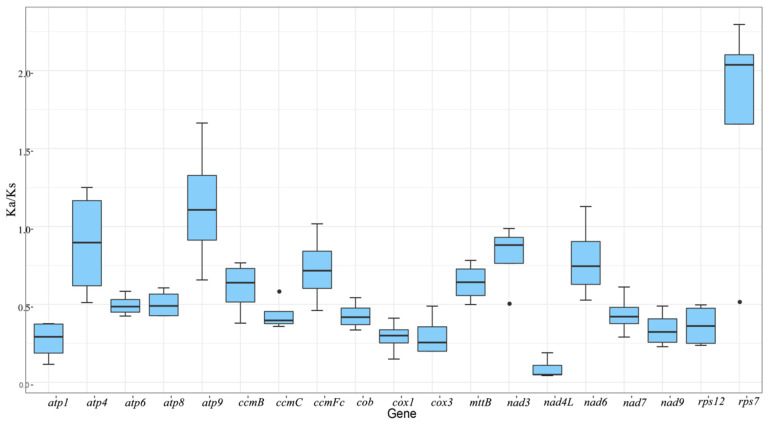
The boxplots of Ka/Ks values among 19 PCGs in the *A. shenzhenica* with four different asparagales. The “*X*” axis shows the name of protein-coding genes, and the “*Y*” axis shows the Ka/Ks values. Black dots represent outliers.

To investigate the evolutionary rate of mitochondrial genes, we calculated the non-synonymous substitution rate (Ka) and synonymous substitution rate (Ks) for the 19 shared PCGs of *A. shenzhenica* against the *Allium cepa*, *Gastrodia elata*, *Hemerocallis citrina*, and *Asparagus officinalis* mitogenomes. As shown in Figure 3, the Ka/Ks ratios were much lower than 1 in the majority of protein-coding genes, indicating the stability of the protein function of these genes during evolution. In contrast, the Ka/Ks ratios of *atp9* (1.13) and *rps7* (1.72) were greater than 1, implying that these genes were subjected to positive selection. In particular, the Ka/Ks ratios of *rps7* in *A. shenzhenica* and *G. elata* were significantly higher than 2(2.3), whereas those of *H. citrina* and *A. officinalis* were also 2.04 (Table S2), indicating that they may be very crucial for the evolution of *A. shenzhenica*. According to previous reports, small mitochondrial subunit proteins encoded by the *rps7* gene are essential for various biological activities in eukaryotes, such as embryonic development, leaf formation, and reproductive tissue formation [19,20,21].

Additionally, the ratio of the *nad3* (0.81) gene was close to 1, indicating that it experienced neutral evolution because of the divergence of *A. shenzhenica* and four other Asparagales from its last common ancestor.

### 2.4. Prediction of RNA Editing Sites in PCGs

RNA editing refers to the process of altering genetic information at the mRNA level, including the deletion, insertion, or replacement of nucleotides. We used the web-based PREP-Mt tool to predict 416 RNA-editing sites and 100% C-to-U RNA editing in 28 PCGS of *A. shenzhenica*. Among them, there were 12 site conversions of CCT to TTT and two conversions of CCC to TTC. Moreover, the proportions were 39.9% (166 sites) for the projected first base location of the codon, 64.3% (255 sites) for the expected second base position, and none for the predicted third base position (Table S3). Instead of the lack of RNA editing at this site, the inadequacies of the PREP-Mt prediction methodology are likely to be responsible for the absence of projected RNA editing sites at the silencing site. Therefore, experimental techniques or proteomic data are required to identify RNA editing in silent editing locations [22,23].

**Figure 4:**
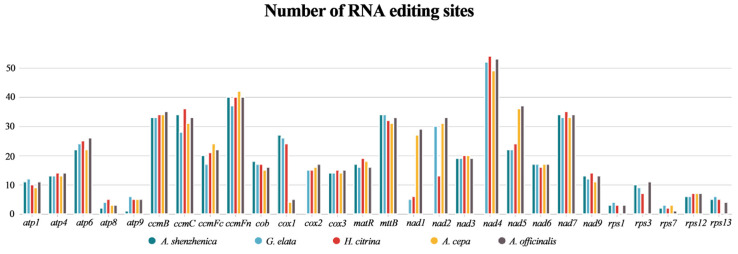
The distribution of RNA editing sites across the 28 PCGs of the mitogenomes of *A. shenzhenica* with four different asparagales.

The number of RNA editing sites varied greatly among the different genes, and the predicted RNA editing sites encoded by complex I (NADH dehydrogenase) and cytochrome c biogenesis genes (*ccmB*, *ccmC*, *ccmFc*, and *ccmFn*) were higher on average (Figure 4). Upon comparing the RNA editing sites of the five asparagine species, we found that the *nad4* gene encoded the most RNA editing sites, whereas *rps7* encoded the least.

### 2.5. Identification of Repeat Sequences

Repetitive sequences consist of simple sequence repeats (SSRs), tandem repeats, and dispersed repeats sequences. A total of 226 SSRs were discovered in *A. shenzhenica* mitogenome, including 57 (25.22%) monomers, 57 (25.22%) dimers, 39 (17.26%) trimers, 61 (26.99%) tetramers, 10 (4.42%) pentamers, and two (0.88%) hexamers. Almost 50% of the repetitions of 226 SSRs were either monomers or dimers. Additional analysis of the repetitive units of the SSRs revealed that G/C only occupied 8.8% of the monomers, whereas A/T accounted for 91.2% of the monomers. The high AT content in *A. shenzhenica* mononucleotide SSRs was consistent with the high AT content (55.6%) of the entire *A. shenzhenica* mitogenome. Table S4 shows the size and position of the hexamers and pentamers, all of which were found in intergenic spacers.

**Figure 5:**
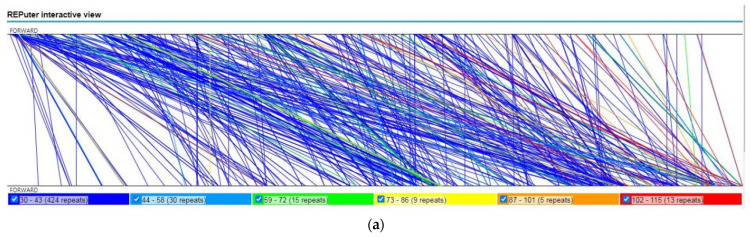

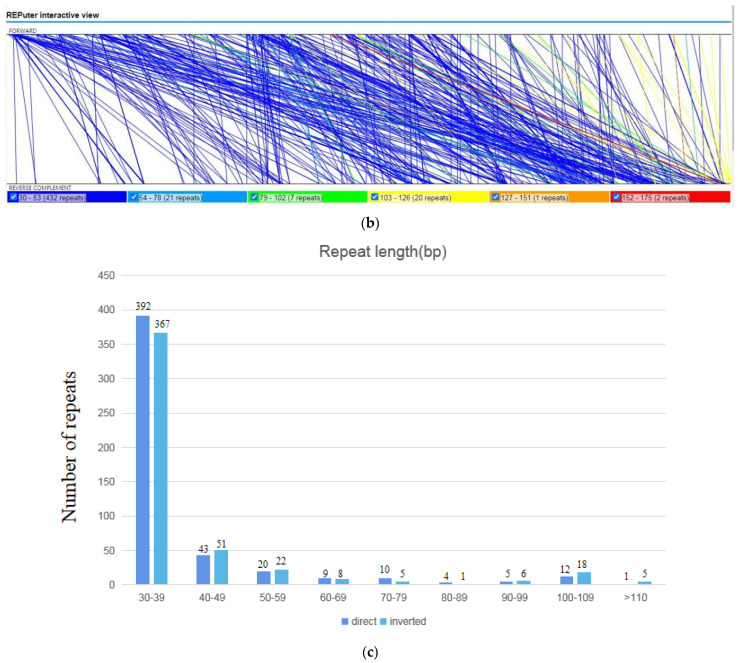
The repeats sequences of *A. shenzhenica* mitogenome. (**a**) The synteny between the mitogenome and its forward copy showing the direct repeats. (**b**) The synteny between the mitogenome and its reverse complimentary copy, showing the inverted repeats. (**c**) The length distribution of reverse and inverted repeats in *A. shenzhenica* mitogenome. The numbers on the horizontal axis of the histogram represents the number of repetitions of designated lengths.

Tandem repeats, also known as satellite DNA, are core repeating units of 1–20 bases that are repeated numerous times. They exist extensively in eukaryotic and certain prokaryotic genomes [24]. The mitogenome of *A. shenzhenica* contained 13 tandem repeats with a matching degree of more than 95% and lengths ranging from 11 to 62 bp (Table S5). Dispersed repeats in *A. shenzhenica* mitogenome were observed using the REPuter program [25]. As a result, 979 repeats with lengths equal to or greater than 30 were found, 496 of which were straight, and 483 of which were inverted. The longest straight repetition was 115 bp, and the largest inverted repeat was 153 bp. Figure 5 shows the length distributions of the straight and inverted repeats. The 30–39 bp repetitions were found to be the most prevalent for both repeat types.

### 2.6. Characterization of A. shenzhenica Cpgenome Transfer into the Mitogenome

**Figure 6:**
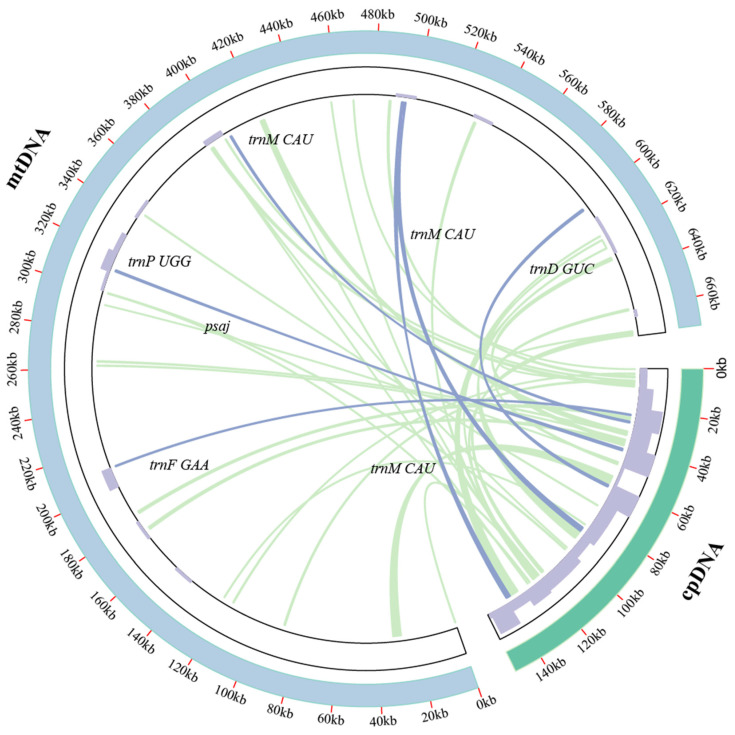
Transfer events in the cpgenome and mitogenome of *A. shenzhenica*. The blue and green outer arcs represent the mitogenome and cpgenome, respectively. Additionally, the inner arcs show the homologous DNA fragments. The scale is shown on the outer arcs, with intervals of 20 kb. The purple bars in the inner circle represent gene density.

The *A. shenzhenica* mitogenome sequence was approximately 4.5 times longer than its cp genome (151,676 bp). The fragments ranged from 30 to 4725 bp in sequence identity with their original cp counterparts. A total of 44 fragments with a length of 34,456 bp migrated from the cp genome to the mitogenome of *A. shenzhenica*, accounting for 5.12% of the mitogenome (Figure 6). Five integrated annotated genes were located on these fragments, including four tRNA genes and one cp genome protein-coding gene, namely, *trnM-CAU*, *trnD-GUC*, *trnP-UGG*, *trnF-GAA*, and *psaj*. Our data also demonstrated that some genes, such as *ycf2*, *accD*, *rrn16*, *psbB*, *rpoB,* etc., migrated from the cp genome to the mitogenome. However, most of them lost their integrity during evolution, and only fragmentary sequences of these genes can presently be found in the mitogenome (Table 3). Through the chloroplast transfer event segments, we found that most tRNA genes were much more conserved than protein-coding genes, probably because they play important roles in the *A. shenzhenica* mitogenome.

### 2.7. Phylogenetic Analysis and Gene Loss of Monocotyledon Mitogenomes

**Figure 7:**
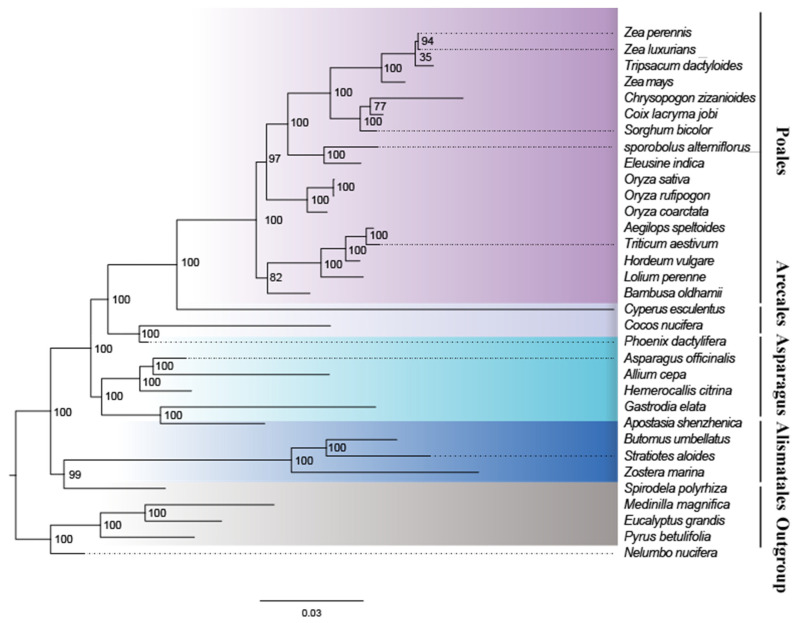
Maximum likelihood phylogenies of *A. shenzhenica* within 28 monocots species. The maximum likelihood tree was constructed based on the mitochondrial sequences of 28 conserved protein-coding genes. Colors indicate the families that the specific species belongs to.

To understand the evolution of *A. shenzhenica* and the monocot mitogenome, we performed phylogenetic analyses on *A. shenzhenica* and 28 other monocots and four dicots (designated as the outgroup). Table S6 lists the accession numbers for the mitogenomes analysed in this study. A phylogenetic tree was constructed using an aligned data matrix comprising 28 conserved protein-coding genes from these species, as illustrated in Figure 7. The phylogenetic tree strongly supported the separation of monocots and dicotyledonous plants. Moreover, taxa from four orders (Alismatales, Asparagus, Arecales, and Poales) were well clustered. The clustering relationships of taxa in the phylogenetic tree in this study were consistent with those of previous studies examining the evolutionary relationships of these species.

As shown in Figure 7, the bootstrap values of most nodes were supported by more than 70%, and 25 nodes were supported by 100%. According to the maximum likelihood (ML) tree, *A. shenzhenica* and *G. elata* were classified into one clade (orchid) with a 100% bootstrap value, whereas this clade formed a sister relationship with the other four Asparagales. From the base group upward, the value for the separation of Alismatales from the clade composed of Asparagus, Arecales, and Poales was 100%. The bootstrap value for the separation of Asparagus and Arecales was 100%, and that of the separation of Arecales from the clade composed of Poales was also 100%.

**Figure 8:**
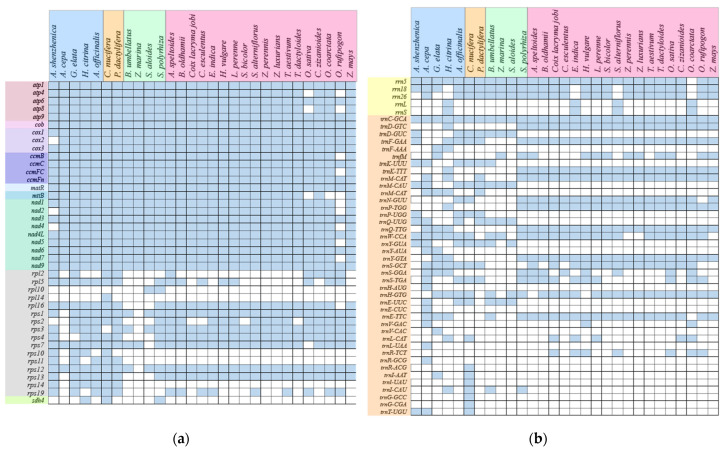
Comparison of genes contents between of *A. shenzhenica* and other 28 monocots species mitogenome. (**a**). The genes of CDS. (**b**). The genes of RNA. Blue-colored boxes represent active genes and blank boxes represent gene loss.

Furthermore, we compared the *A. shenzhenica* with 28 other monocots mitogenomes and discovered that genes composition of monocots mitogenomes differed. As illustrated in Figure 8a, the majority of mitochondrial protein-coding and rRNA genes are highly conserved, *cox2* is only lost in the *A. shenzhenica*, and *rpl10*, *rpl14,* and *sdh4* are lost in most monocots. In the evolutionary history of monocots, a considerable number of mitochondrial-derived tRNAs loss events occurred; only *trnC-GCA* is conservative (Figure 8b). However, most of the tRNAs in Poales are regularly retained.

## 3. Discussion

### 3.1. Characterization of the A. shenzhenica Mitogenome

Most orchids exist as epiphytes in the wild. Orchid roots, stems, leaves, flowers, and seeds have adapted to a wide variety of habitat environments and have evolved unique structures and functions. The advent of rapid and cost-effective genome-sequencing technologies has accelerated our understanding of plant evolution. Mitochondria are the power stations of plants that produce the energy required to carry out life processes. Plant mitogenomes are more complex than the chloroplast genome because of factors, such as complex structures and size variations [10,18]. Our study is the first to report the *A. shenzhenica* mitogenome and its characterization. Compared with other species of Asparagales, the mitogenome size of *A. shenzhenica* is moderate. However, the mitogenome size is remarkably smaller than that of *G. elata* [10], but larger than those of *A. cepa*, *A. officinalis*, and *H. citrina* [26,27,28]. The overall GC content is 44.4%, which is similar to that of other species of Asparagales (42.9–46.7%) (Table 4).

Most sequences in the *A. shenzhenica* mitogenome are non-coding, and protein-coding genes account for 4.21%. Compared to most mitogenomes, protein-coding genes are 2.45% in *G. elata*, 3.31% in *C. comosum*, 4.70% in *A. cepa*, 6.45% in *A. officinalis*, and 8.27% in *H. citrina*, which is mainly due to the frequent recombination of repeated sequences in the mitogenome and the integration of foreign sequences during evolution.

### 3.2. Repeat Sequences, Ka/Ks, and RNA Editing Sites Length of Protein Coding Region (%)

Mitochondrial repeat sequences are essential for intermolecular recombination because they can contribute to extreme mitogenome sizes and structural differences [29,30]. The mitogenome of *A. shenzhenica* contains 113 repeat sequences longer than 50 bp, accounting for 1.38% of its genome. The maximum length is 175 bp and does not contain medium or large repeats. We suspect that recombination is less frequent in the *A. shenzhenica* mitochondrion. Moreover, repeats are poorly conserved in plant mitogenomes, even within the same family. As shown in Table 4, the total length of the repeats ranges from 9298 bp (1.38%) in *A. shenzhenica* to 310,943 bp (57.95%) in *A. cepa*. Interestingly, the mitogenome of *A. cepa* is 536,617 bp, and that of *A. shenzhenica* is 672,872 bp, suggesting that the size of the genome is determined not only by the expansion of repeated sequences, but also by other factors. This mtDNA heteroplasmy may provide more genetic resources for evolutionary selection [31], imparting ecological and genetic fitness to *A. shenzhenica* during its evolution.

Ka/Ks analysis and comparison of mitogenome features provide a comprehensive understanding of plant mitogenome evolution. In the present study, *atp4*, *atp9*, *ccmFc*, and *nad6* undergo positive selection during evolution. Different plant species under conditions involving positive selection pressure during evolution, including *atp8*, *ccmFn*, *matR*, *ccmB* and *mttB*, which have also been reported [5,7,22,32]. However, the *A. shenzhenica* mitogenome is conserved, and most of the PCGs have undergone neutral and negative selection compared to other Asparagales species. In general, most of the results of this investigation are consistent with the previous reports. In addition, *nad4L* shows the lowest Ka/Ks ratio among *A. shenzhenica* mitochondrial genes. The *nad4L* Ka/Ks ratio is < 0.5 in different species [33]. There are two copies of *nad4L* in *A. shenzhenica*, and we suspect that this gene plays an important role in *A. shenzhenica*.

RNA editing, a post-transcriptional mechanism that occurs in higher plant chloroplasts and mitogenomes, aids in protein folding [34,35]. Each organelle is highly lineage-specific in terms of the frequency and the type of RNA editing [36,37,38]. In the earlier studies, *Oryza sativa* exhibited 491 RNA editing sites in 34 genes [39], and *Phaseolus vulgaris* showed 486 RNA editing sites in 31 genes [40]. In the present study, we predicted RNA editing sites in 28 PCGs common to *G. elata*, *H. citrina*, *A. cepa*, and *A. officinalis* mitogenomes. As shown in Figure 4, the number of RNA editing sites predicted in mitogenomes of different Asparagales species is very conserved, from 416 sites in *A. shenzhenica* to 552 sites in *Asparagus officinalis*. Of these, 514 are found in *G. elata*, of which 347 are shared with *A. shenzhenica*. These results suggest that they share highly conserved PCGs.

### 3.3. DNA Fragment Transfer Events

Intracellular gene transfer between different genomes (mitochondria, nuclei, and chloroplasts) has been widely examined via sequencing analyses [41,42]. Prior studies have found high levels of nuclear DNA translocation to organelles in monocots [43,44]. Nuclear NDH complex-related genes are lost, along with cp-ndh genes in orchids [45]. However, the cp-ndh gene in *A. shenzhenica* is not completely lost, and only *ndhH*, *ndhE*, *ndhF*, and *ndhG* are lost; whether it is transferred to a nuclear gene is unknown.

Horizontal gene transfer from chloroplasts to mitochondria has been reported several times. However, the length and number of transfer fragments vary significantly between species [7]. In our study, we identified 44 fragments that have been transferred from the cp genome (22.72% of the cp genome) to the mitogenome. These included five integrated genes, (four tRNA genes and one protein-coding gene, *psaJ*). Interestingly, we found that chloroplast transfer fragments were randomly scattered across every region of the chloroplast and not just in the repetitive regions [7].

### 3.4. Phylogenetic Analyses

A phylogenetic tree based on the entire mitochondrial protein-coding gene sequence was constructed to explore the evolutionary relationships between mitochondria in monocots. Phylogenetic analysis using the mitogenome shows results congruent with those of Petersen [4]. The evolutionary relationships among the four classes of monocots lineages are well resolved in this study. Our study supports mitogenomics as an informational tool to address the systematic relationships among families, orders, or higher taxonomic levels of angiosperms. However, the phylogenetic differentiation of some nodes, such as Zea, *Tripsacum dactyloides*, *chrysopogon zizanioides*, and *coix lacrymajobi* is not well resolved in the present analysis. This suggests that they may have a close genetic relationship or ancestral relationship. An extended sampling of more representative monocots plants, as well as comparisons of mitogenome phylogenetic with plastid DNA, nuclear DNA, and morphology data, are necessary to confidently establish the phylogenetic relationships of monocots.

## 4. Materials and Methods

### 4.1. Plant Materials and Sequencing

We obtained plant materials of *A. shenzhenica* from the Shenzhen, Guangdong Province, China. A total DNA of *A. shenzhenica* was isolated as previously reported [46]. mtDNA was extracted from purified mitochondria using the cetyltrimethylammonium bromide technique [47]. For *A. shenzhenica*, a 250-bp paired-end library and a 3-kb mate-pair library were produced and sequenced on an Illumina HiSeq 2500 platform using two alternative techniques. Trimmomatic v0.36 was used to remove low-quality bases and adaptor sequences from the raw Illumina reads [48].

### 4.2. Mitogenome Assembly and Annotation

We used SPAdes v3.10.1 for de novo assembly of the *A. shenzhenica* mitogenome [49]. Furthermore, we ran many SPAdes runs with different k-mer values (k = 77, 101, and 127) and utilized QUAST [50] to evaluate and select 127 as the optimal k-mer number for multiple assembly. Finally, we identified only one candidate mitochondrial scaffold, which can be mapped as a circular molecule with a pair of direct repeats at its both ends. Sanger sequencing was used to confirm the connector and filled the seven remaining gaps in this scaffold.

The Geneious [51] program was used to predict mitochondrial protein-coding genes. tRNAscan-SE v1.21 [52] and RNAmmer 1.2 Server [53] were used to identify the tRNA and rRNA genes, respectively. The start/stop codons and exon-intron boundaries of genes were manually corrected. ORFfinder (https://www.ncbi.nlm.nih.gov/orffinder/, accessed on 30 June 2022) was used to examine ORFs longer than 300 bp inside intergenic sequences, and BlastN [54] was used to identify repetitions with more than 95% identity in each mitogenome. We used a shell script to analyze the Guanine-Cytosine (GC) content and Circos v0.69 to show the circular physical map of all mitogenomes [55]. The RNAweasel program was used to predict Group II introns.

In codon usage analysis, codonW v1.4.4 was used to determine relative synonymous codon use (RSCU) of selected protein-coding genes in the mitogenome [56]. Then, the R package ggplot2 was used for plotting.

### 4.3. Selective Pressure Analysis

The nonsynonymous (Ka) and synonymous (Ks) substitution rates of each PCG between *A. cepa*, *G. elata*, *H. citrina*, and *A. officinalis* were estimated. MEGA 6.0 was used to separately align orthologous gene pairs. DnaSP v5.10 was used to calculated Ka, Ks, and Ka/Ks values [57]. The ggplot2 v3.3.6 was used to generate a boxplot of paired Ka/Ks values [58].

### 4.4. Prediction of RNA Editing Sites

The online PREP-Mt server suite (http://prep.unl.edu/, accessed on 10 October 2022) was used to anticipate potential RNA editing sites in *A. shenzhenica* PCGs and the other four Asparagales mitogenomes (*A. cepa*, *A. officinalis*, *G. elata*, and *H. citrina*). The cutoff value was set to 0.2 [23] to produce a more accurate prediction. A lower cut-off value predicts more real edit sites, but it increases the likelihood of misidentifying an unedited site as an edited one.

### 4.5. Analysis of Repeat Structure and Sequence

The MISAv2.1 (https://webblast.ipk-gatersleben.de/misa/, accessed on 1 September 2022) was used to assess the simple sequence repeats (SSRs) of the *A. shenzhenica* mitogenome [59], with the motif size of one to six nucleotides and thresholds of 10, 5, 4, 3, 3 and 3, respectively. Tandem Repeats Finder v4.09 program [24] (http://tandem.bu.edu/trf/trf.submit.options.html, accessed on 1 September 2022) with default parameters was used to find tandem repeats with >6 bp repeat unit. Using the REPuter web server [25] (https://bibiserv.cebitec.uni-bielefeld.de/reputer, accessed on 5 September 2022) with the parameters “Hamming Distance 3, Maximum Computed Repeats 5000, Minimum Repeat Size 30,” dispersed repeats including forward, reverse, palindromic, and complementary repeats were discovered.

### 4.6. DNA Transfer between the Chloroplast and the Mitochondrion

The *A. shenzhenica* cp genome (MG772639.1) was obtained from the NCBI Organelle Genome Resources database. BLASTN was used to analyze sequence similarity between the cpgenome and the mitogenome in order to detect transferred DNA fragments, and the e-value cut-off was 1 × 10^−5^ [60]. The Circos module implemented in TBtools v1.105 was used to visualize the results [61,62,63].

### 4.7. Evaluating the Phylogenetic Mitochondrial Protein-Coding Genes

We downloaded the mitochondrial protein coding gene sequences of 29 different monocots and four dicotyledons and exported them as FASTA model files. Then, using the annotation module of the Geneious Prime v2022.0.1 platform, the CDS sequences of mitochondrial genes of 33 species were selected, and the exported the data as an excel sheet. Subsequently, the protein coding genes of 28 core protein coding genes (*atp1*, *atp4*, *atp6*, *atp8*, *atp9*, *ccmB*, *ccmC*, *ccmFc*, *ccmFn*, *cob*, *cox1*, *cox2*, *cox3*, *matR*, *nad1*, *nad2*, *nad3*, *nad4*, *nad4L*, *nad5*, *nad6*, *nad7*, *nad9*, *rps1*, *rps3*, *rps7*, *rps12*, and *rps13*) were extracted from the mitogenomes of 33 species. Four species (*Eucalyptus grandis*, *Medinella magnetica*, *Nelumbo nucifera,* and *Pyrus betulifolia*) were used as outgroups. The protein-coding genes of 33 species were aligned by MEGA7.

Using the Concatenate Sequence module of Phylosuite v1.1.16 platform (ref), we converted the protein coding gene matrix into PHY tree format file. The (Maximum Likelihood, ML) phylogenetic tree was reconstructed using RAxML-HPC2 on XSEDE 8.2.12 in CIPRES science Gateway V 3.3 [64]. The ML tree was constructed by using the nucleotide replacement model GTRCAT, using the Bootstrap algorithm, with repeated calculation at 1000 times, and the other parameters were set to default [65]. The MP tree was constructed by heuristic search and the branch exchange algorithm (Tree-Bisection-Reconnection, TBR). All nucleotide characters are equally weighted, and the search was carried out by the method of arbitrary repetition of 1000 times. The reliability of the phylogenetic tree was analyzed by the bootstrap method of 1000 repetitions [66].

## 5. Conclusions

In this study, we assembled and annotated the *A. shenzhenica* mitogenome and performed a comprehensive analysis based on the annotated sequences. The draft mitogenome of *A. shenzhenica* was circular, with a length of 672,872 bp, and it comprised 54 genes, including 36 protein-coding genes, 16 tRNA genes, and two rRNA genes. Upon comparing the mitogenome and cp genome sequences, we discovered 44 fragments that were transferred from the cp genomes to the mitochondrial sequences. Furthermore, we analysed the *A. shenzhenica* mitogenome for codon usage, sequence repeats, RNA editing, and selection pressure. Moreover, the evolutionary status of the monocots was identified by phylogenetic analysis of the mitogenomes of *A. shenzhenica* and 28 other monocots. This study provides new insights into the diversity and evolution of orchid mitogenomes. We demonstrate mitochondrial evidence for *A. shenzhenica* and provide important insights into the evolution of orchids and monocots.

## Figures and Tables

**Table 1:** Genes present in the mitogenome of *A. shenzenica*.

Group of Genes	Name	Length	Start Codon	Stop Codon	Amino Acids
Complex V (ATP synthase)	*atp1*	1524	ATG	TGA	507
	*atp4*	588	ATG	TAA	195
	*atp6*	1194	ATG	TAA	398
	*atp8*	474	ATG	TAA	157
	*atp9*	225	ATG	TAA	75
Cytochrome c biogenesis	*ccmB*	621	ATG	TGA	206
	*ccmC*	762	ATG	TGA	254
	*ccmFc* *	1389	ATG	TAA	463
	*ccmFn*	1857	ATG	TAA	619
Complex III (ubiquinol cytochrome c reductase)	*cob*	1173	ATG	TAG	390
Complex IV (cytochrome c oxidase)	*cox1*	1584	ATG	TAA	527
	*cox3*	798	ATG	TGA	265
Maturases	*matR*	1926	ATG	TAG	641
Transport membrane protein	*mttB*	453	TTG	TAA	150
Complex I (NADH dehydrogenase)	*nad1*	384	ATT	TAG	128
	*nad3*	360	ATG	TAA	120
	*nad4L(2)*	264	ATG	TAA	88
	*nad5* *	1452	ATG	TGA	483
	*nad6*	618	ATG	TAA	205
	*nad7* ****	1185	ATG	TAG	392
	*nad9*	573	ATG	TAA	190
Ribosomal proteins (LSU)	*rpl2* *	1572	ATG	TAG	522
	*rpl5*	555	ATG	TAA	184
	*rpl16*	558	ATG	TAA	185
Ribosomal proteins (SSU)	*rps1*	525	ATG	TAA	174
	*rps3* *	1743	ATG	TAG	579
	*rps4*	1032	ATG	TAA	343
	*rps7*	447	ATG	TAA	149
	*rps10* *	339	ATG	TAA	113
	*rps11*	516	ATG	TAA	172
	*rps12*	378	ATG	TGA	126
	*rps13*	351	ATG	TGA	116
	*rps14*	324	ATG	TAG	107
	*rps19*	561	ATG	TGA	186
Ribosomal RNAs	*rrn5*	117	-	-	-
	*rrn18*	1994	-	-	-
Transfer RNAs	*tRNA-Phe*	73	-	-	-
	*tRNA-Lys*	75	-	-	-
	*tRNA-Gln*	71	-	-	-
	*tRNA-Thr*	71	-	-	-
	*tRNA-Met ^a^*	74	-	-	-
	*tRNA-Cys*	73	-	-	-
	*trnC-GCA*	71	-	-	-
	*trnD-GUC ^a^*	74	-	-	-
	*trnF-GAA ^a^*	73	-	-	-
	*trnK-UUU*	73	-	-	-
	*trnM-CAU ^a^*	73	-	-	-
	*trnP-UGG ^a^*	74	-	-	-
	*trnQ-UUG*	72	-	-	-
	*trnQ-UUG*	72	-	-	-
	*trnW-CCA*	74	-	-	-
	*trnY-GUA*	83	-	-	-

Note: Numbers after gene names are the number of copies. The superscripts * and **** represent one and four introns contained, respectively. The superscripts *a* indicates the chloroplast-derived genes.

**Table 2:** Relative synonymous codon usage and codon numbers of *A. shenzhenica* mitochondrial PCGs.

AA	Codon	No.	RSCU	AA	Codon	No.	RSCU
Phe	UUU	276	0.92	Ser	UCU	196	1.45
	UUC	327	1.08		UCC	145	1.07
Leu	UUA	135	0.71		UCA	186	1.38
	UUG	233	1.23		UCG	140	1.04
	CUU	233	1.23	Pro	CCU	95	0.75
	CUC	172	0.91		CCC	95	0.75
	CUA	201	1.06		CCA	190	1.5
	CUG	166	0.87		CCG	126	1
Ile	AUU	185	0.96	Thr	ACU	76	0.88
	AUC	205	1.06		ACC	83	0.97
	AUA	189	0.98		ACA	98	1.14
Met	AUG	214	1		ACG	87	1.01
Val	GUU	133	0.97	Ala	GCU	92	1.14
	GUC	141	1.03		GCC	64	0.8
	GUA	132	0.96		GCA	90	1.12
	GUG	142	1.04		GCG	76	0.94
Tyr	UAU	133	0.93	Cys	UGU	115	0.95
	UAC	154	1.07		UGC	128	1.05
Ter	UAA	156	0.81	Ter	UGA	199	1.03
	UAG	226	1.17	Trp	UGG	218	1
His	CAU	120	0.98	Arg	CGU	56	0.55
	CAC	126	1.02		CGC	58	0.57
Gln	CAA	159	0.99		CGA	117	1.14
	CAG	161	1.01		CGG	125	1.22
Asn	AAU	160	1.1	Ser	AGU	62	0.46
	AAC	131	0.9		AGC	81	0.6
Lys	AAA	245	1.1	Arg	AGA	168	1.64
	AAG	200	0.9		AGG	91	0.89
Asp	GAU	118	1.22	Gly	GGU	104	0.89
	GAC	76	0.78		GGC	57	0.49
Glu	GAA	227	1.12		GGA	163	1.39
	GAG	178	0.88		GGG	144	1.23

9379 codons in *A. shenzhenica* (used Universal Genetic code).

**Table 3:** Fragments transferred from chloroplast to mitochondria in *A.shenzhenica*.

No	Alignment Length	% Identity	Gap Opens	CP Start	CP End	Mt Start	Mt End	Gene
1	4725	98.836	11	132,666	137,368	626,018	621,308	*ycf2 ^a^*
2	4725	98.836	11	88,264	92,966	621,308	626,018	*ycf2 ^a^*
3	3976	99.623	3	56,121	60,093	28,471	32,446	*-*
4	2006	97.906	13	32,120	34,125	421,999	420,022	*accD ^a^*
5	2081	94.522	14	86,296	88,371	495,204	493,213	*trnM-CAU*, *rpl23 ^a^*
6	2081	94.522	14	137,261	139,336	493,213	495,204	*trnM- CAU*, *rpl23 ^a^*
7	1373	97.815	3	36,982	38,349	631,371	632,733	*petA ^a^*
8	1242	99.758	0	7898	9139	391,287	392,528	*rpoC2 ^a^*
9	1219	99.918	0	116,093	117,311	670,487	671,705	*rrn23 ^a^*
10	1076	92.937	11	24,885	25,947	184,256	183,219	*ndhc ^a^*
11	746	100	0	121,777	122,522	671,706	672,451	*rrn16 ^a^*
12	685	98.102	1	49,035	49,719	659,617	658,939	*psbB ^a^*
13	704	95.313	3	3891	4589	487,500	486,810	*rpoB ^a^*
14	731	94.391	14	38,847	39,548	174,249	173,520	*psbj ^a^*
15	924	87.229	19	61,059	61,968	602,745	603,612	*trnD-GUC*
16	616	93.831	9	26,940	27,551	402,787	403,382	*trnM-CAU*
17	485	89.691	4	6392	6871	88,407	87,960	*rpoCI ^a^*
18	461	89.588	12	41,276	41,728	311,079	311,516	*trnP-UGG*
19	382	93.455	6	38,370	38,751	174,332	174,696	*-*
20	355	92.113	0	33,561	33,915	264,149	264,503	*accD ^a^*
21	263	94.677	1	2518	2780	116,752	116,501	*rpoB ^a^*
22	260	93.462	3	6067	6325	400,045	399,792	*rpoCI ^a^*
23	196	97.449	0	72,669	72,864	343,153	343,348	*psaA ^a^*
24	887	74.183	37	121,923	122,786	531,470	532,328	*rrn18 ^b^*
25	179	97.207	1	42,023	42,201	311,569	311,746	*psaj*
26	196	93.367	1	36,503	36,689	262,195	262,000	*cemA ^a^*
27	311	83.28	10	23,453	23,757	210,056	209,764	*trnF-GAA*
28	148	93.919	1	7335	7482	670,488	670,348	*rpoCI ^a^*
29	111	99.099	0	125,099	125,209	299,455	299,345	*-*
30	111	99.099	0	100,423	100,533	299,345	299,455	*rps12 ^a^*
31	105	100	0	119,735	119,839	105	1	*trnA ^a^*
32	105	100	0	119,163	119,267	420,021	419,917	*trnA ^a^*
33	105	100	0	120,794	120,898	457,200	457,304	*trnI ^a^*
34	105	100	0	121,777	121,881	619,103	618,999	*rrn16 ^a^*
35	129	93.023	1	100,558	100,679	299,332	299,204	*rps12 ^a^*
36	129	93.023	1	124,953	125,074	299,204	299,332	*-*
37	127	89.764	3	84,364	84,484	121,858	121,984	*pabA ^a^*
38	92	85.87	5	99,245	99,332	392,626	392,537	*rps7 ^a^*
39	92	85.87	5	126,300	126,387	392,537	392,626	*rps7 ^a^*
40	50	100	0	270	319	468,564	468,613	*-*
41	50	96	0	38,799	38,848	174,326	174,277	*-*
42	41	97.561	0	100,397	100,437	299,481	299,441	*rps12 ^a^*
43	41	97.561	0	125,195	125,235	299,441	299,481	*-*
44	30	100	0	48,483	48,512	293,196	293,167	*psbB ^a^*
Total	34,456							

Notes: Lowercase *a* indicates the partial sequence found in mitogenome. Lowercase *b* indicates the mt-derived genes.

**Table 4:** Summary of features in *A. shenzhenica* and other asparagopsis mitogenomes.

Feature	*A. shenzhenica*	*G. elata*	*A. cepa*	*A. officinalis*	*H. citrina*
Genome size (bp)	672,872	1,339,825	536,617	492,062	468,462
GC content (%)	44.4	44.6	42.9	43.4	45.2
Length of protein coding region (%)	28,332 (4.21%)	32,801 (2.45%)	25,203 (4.70%)	31,749 (6.45%)	38,758 (8.27%)
Length of tRNA genes (%)	1176 (0.17%)	1479 (0.11%)	1871 (0.35%)	1284 (0.26%)	1282 (0.27%)
Length of rRNA genes (%)	2111 (0.31%)	2085 (0.16%)	5100 (0.95%)	10,981 (2.23%)	7300 (1.56%)
Number of protein coding genes	36	39	27	36	45
Number of rRNA genes	2	3	3	6	4
Number of tRNA genes	16	20	25	17	17
Total genes	54	62	55	59	66
Length of >50 repeats (%)	9298 (1.38%)	127,244 (9.50%)	310,943 (57.95%)	50,419 (10.25%)	132,110 (28.20%)
Longest repeat (bp)	175	1773	31,800	12,365	16,460
Accession Numbers	OQ645347	MF070084-102	AP018390	MT483944	MZ726801-3

## Data Availability

The entire complete mitogenome sequence with gene annotation has been submitted in the NCBI GenBank under the accession number OQ645347. The sequence data utilized in this study can be found in Supplementary Materials.

## Supplementary Materials

The following supporting information can be downloaded at: https://www.mdpi.com/article/10.3390/ijms24097837/s1.
