# Supplementary material for: Apostasia Mitochondrial Genome Analysis and Monocot Mitochondria Phylogenomics
Source: Int J Mol Sci. 2023 Apr 25;24(9):7837. doi: 10.3390/ijms24097837 (PMC10178136; doi:10.3390/ijms24097837)
Supplement: Supplementary file 1 [file ijms-24-07837-s001.zip › ijms-2347460-supplementary.pdf]

Apostasia mitochondrial genome analysis and monocot mitochondria phylogenomics

Shijie Ke[1], Ding-Kun Liu[1], Xiong-De Tu[1], Xin He[1], Meng-Meng Zhang[1], Meng-Jia Zhu[1], Diyang Zhang[2], Cuili Zhang[2], Siren Lan[1,2*] and Zhong-Jian Liu[2*]

[1]Fujian Colleges and Universities Engineering Research Institute of Conservation and Utilization of Natural Bioresources, College of Forestry, Fujian Agriculture and Forestry University, Fuzhou, 350002, China
[2]Key Laboratory of Orchid Conservation and Utilization of National Forestry and Grassland Administration at College of Landscape Architecture and Art, Fujian Agriculture and Forestry University, Fuzhou, 350002, China

3210422027@fafu.edu.cn (S.K.); fjliudk@163.com (D.-K.L.); tttxd163@163.com (X.-D.T); 5220422102@fafu.edu.cn (X.H.); 1220428020@fafu.edu.cn (M.-M.Z.); 1200455008@fafu.edu.cn (M.-J.Z.); diyangzhang@126.com (D.Z.); cuilizhang@fafu.edu.cn (C.Z.).

*Correspondence: zjliu@fafu.edu.cn; lkzx@fafu.edu.cn;

Supplementary Tables

Table S1 Gene content of *A.shenzhenica* mitogenome.

Group of Genes	Gene Name
Complex I (NADH dehydrogenase)	*nad1, nad3, nad4L*(2), *nad5**, *nad6, nad7*****, *nad9*
Complex III (ubiquinol cytochrome c reductase)	*cob*
Complex IV (cytochrome c oxidase)	*cox1, cox3*
Complex V (ATP synthase)	*atp1, atp4, atp6, atp8, atp9*
Cytochrome c biogenesis	*ccmB, ccmC, ccmFc**, *ccmFn*
Ribosomal proteins (SSU)	*rps1, rps3**, *rps4, rps7, rps10**, *rps11, rps12, rps13, rps14, rps19*
Ribosomal proteins (LSU)	*rpl2**, *rpl5, rpl16*
Maturases	*matR*
Transport membrane protein	*mttB*
Ribosomal RNAs	*rrn5, rrn18*
Transfer RNAs	*trnD-GUC, trnA-Met, trnQ-UUG, trnM-CAU, trnK-UUU, trnQ-UUG, trnP-UGG, trnW-CCA, trnA-Cys, trnF-GAA, trnA-Phe, trnA-Lys, trnA-Gln, trnA-Thr, trnY-GUA, trnC-GCA*
Cp transfer proteins	*psaJ*

*: represents intron number; Gene(2): Number of copies of multi-copy genes.

Table S2. Ka/Ks analysis of *A.shenzhenica* and four different Asparagales genes;

Seq1	Seq2	Gene	Ks	Ka	Ka/Ks
Apostasia shenzhenica	*Allium cepa*	*atp1*	0.1098	0.0232	0.2113
Apostasia shenzhenica	*Gastrodia elata*	*atp1*	0.0497	0.0187	0.376257545
Apostasia shenzhenica	*Hemerocallis citrina*	*atp1*	0.0762	0.0088	0.115485564
Apostasia shenzhenica	*Asparagus officinalis*	*atp1*	0.0646	0.0241	0.373065015
Apostasia shenzhenica	*Allium cepa*	*atp4*	0.0415	0.0519	1.25060241
Apostasia shenzhenica	*Gastrodia elata*	*atp4*	0.1157	0.0592	0.511668107
Apostasia shenzhenica	*Hemerocallis citrina*	*atp4*	0.0232	0.0264	1.137931034
Apostasia shenzhenica	*Asparagus officinalis*	*atp4*	0.0351	0.023	0.655270655
Apostasia shenzhenica	*Allium cepa*	*atp6*	0.0808	0.0415	0.513613861
Apostasia shenzhenica	*Gastrodia elata*	*atp6*	0.1093	0.0483	0.441903019
Apostasia shenzhenica	*Hemerocallis citrina*	*atp6*	0.0613	0.0279	0.455138662
Apostasia shenzhenica	*Asparagus officinalis*	*atp6*	0.0613	0.0356	0.580750408
Apostasia shenzhenica	*Allium cepa*	*atp8*	0.5999	0.2566	0.427737956
Apostasia shenzhenica	*Gastrodia elata*	*atp8*	0.1729	0.1048	0.606130711
Apostasia shenzhenica	*Hemerocallis citrina*	*atp8*	0.2326	0.1286	0.552880482
Apostasia shenzhenica	*Asparagus officinalis*	*atp8*	0.314	0.1342	0.427388535
Apostasia shenzhenica	*Allium cepa*	*atp9*	0.0634	0.1055	1.664037855
Apostasia shenzhenica	*Gastrodia elata*	*atp9*	0.1105	0.0726	0.657013575
Apostasia shenzhenica	*Hemerocallis citrina*	*atp9*	0.0866	0.1053	1.215935335
Apostasia shenzhenica	*Asparagus officinalis*	*atp9*	0.086	0.0858	0.997674419
Apostasia shenzhenica	*Allium cepa*	*ccmb*	0.1074	0.0408	0.379888268
Apostasia shenzhenica	*Gastrodia elata*	*ccmb*	0.0754	0.0578	0.766578249
Apostasia shenzhenica	*Hemerocallis citrina*	*ccmb*	0.0646	0.0362	0.560371517
Apostasia shenzhenica	*Asparagus officinalis*	*ccmb*	0.0504	0.0362	0.718253968
Apostasia shenzhenica	*Allium cepa*	*ccmc*	0.0768	0.0447	0.58203125
Apostasia shenzhenica	*Gastrodia elata*	*ccmc*	0.0892	0.0368	0.412556054
Apostasia shenzhenica	*Hemerocallis citrina*	*ccmc*	0.0641	0.023	0.358814353
Apostasia shenzhenica	*Asparagus officinalis*	*ccmc*	0.0704	0.0269	0.382102273
Apostasia shenzhenica	*Allium cepa*	*ccmfc*	0.1076	0.0496	0.460966543
Apostasia shenzhenica	*Gastrodia elata*	*ccmfc*	0.0813	0.0827	1.017220172
Apostasia shenzhenica	*Hemerocallis citrina*	*ccmfc*	0.0641	0.0502	0.783151326
Apostasia shenzhenica	*Asparagus officinalis*	*ccmfc*	0.0731	0.0475	0.649794802

Apostasia shenzhenica	*Allium cepa*	*cob*	0.051	0.0277	0.543137255
Apostasia shenzhenica	*Gastrodia elata*	*cob*	0.0506	0.023	0.454545455
Apostasia shenzhenica	*Hemerocallis citrina*	*cob*	0.0512	0.0172	0.3359375
Apostasia shenzhenica	*Asparagus officinalis*	*cob*	0.0511	0.0195	0.381604697
Apostasia shenzhenica	*Allium cepa*	*cox1*	0.1136	0.0326	0.286971831
Apostasia shenzhenica	*Gastrodia elata*	*cox1*	0.0605	0.0189	0.312396694
Apostasia shenzhenica	*Hemerocallis citrina*	*cox1*	0.0596	0.0089	0.149328859
Apostasia shenzhenica	*Asparagus officinalis*	*cox1*	0.0631	0.026	0.412044374
Apostasia shenzhenica	*Allium cepa*	*cox3*	0.1029	0.0205	0.199222546
Apostasia shenzhenica	*Gastrodia elata*	*cox3*	0.0632	0.0309	0.488924051
Apostasia shenzhenica	*Hemerocallis citrina*	*cox3*	0.0589	0.0184	0.312393888
Apostasia shenzhenica	*Asparagus officinalis*	*cox3*	0.0753	0.015	0.199203187
Apostasia shenzhenica	*Allium cepa*	*mttb*	0.0373	0.0292	0.782841823
Apostasia shenzhenica	*Gastrodia elata*	*mttb*	0.0709	0.0354	0.499294781
Apostasia shenzhenica	*Hemerocallis citrina*	*mttb*	0.0278	0.0197	0.708633094
Apostasia shenzhenica	*Asparagus officinalis*	*mttb*	0.0342	0.0197	0.576023392
Apostasia shenzhenica	*Allium cepa*	*nad3*	0.0594	0.0505	0.85016835
Apostasia shenzhenica	*Gastrodia elata*	*nad3*	0.0844	0.0425	0.503554502
Apostasia shenzhenica	*Hemerocallis citrina*	*nad3*	0.0597	0.0544	0.911222781
Apostasia shenzhenica	*Asparagus officinalis*	*nad3*	0.0471	0.0465	0.987261146
Apostasia shenzhenica	*Allium cepa*	*nad4L*	0.165	0.0206	0.12484848
Apostasia shenzhenica	*Gastrodia elata*	*nad4L*	0.1366	0.0723	0.292825768
Apostasia shenzhenica	*Hemerocallis citrina*	*nad4L*	0.165	0.0206	0.12484848
Apostasia shenzhenica	*Asparagus officinalis*	*nad4L*	0.165	0.0206	0.12484848
Apostasia shenzhenica	*Allium cepa*	*nad6*	0.0708	0.0587	0.829096045
Apostasia shenzhenica	*Gastrodia elata*	*nad6*	0.0531	0.0599	1.128060264
Apostasia shenzhenica	*Hemerocallis citrina*	*nad6*	0.1069	0.0564	0.527595884
Apostasia shenzhenica	*Asparagus officinalis*	*nad6*	0.0852	0.0564	0.661971831
Apostasia shenzhenica	*Allium cepa*	*nad7*	0.0264	0.0107	0.40530303
Apostasia shenzhenica	*Gastrodia elata*	*nad7*	0.0389	0.0113	0.290488432
Apostasia shenzhenica	*Hemerocallis citrina*	*nad7*	0.0157	0.0096	0.611464968
Apostasia shenzhenica	*Asparagus officinalis*	*nad7*	0.0192	0.0084	0.4375
Apostasia shenzhenica	*Allium cepa*	*nad9*	0.075	0.0285	0.38
Apostasia shenzhenica	*Gastrodia elata*	*nad9*	0.0611	0.0299	0.489361702
Apostasia shenzhenica	*Hemerocallis citrina*	*nad9*	0.0704	0.0161	0.228693182

Apostasia shenzhenica	*Asparagus officinalis*	*nad9*	0.0706	0.0188	0.266288952
Apostasia shenzhenica	*Allium cepa*	*rps7*	1.1904	0.6126	0.514616935
Apostasia shenzhenica	*Gastrodia elata*	*rps7*	0.0287	0.0659	2.296167247
Apostasia shenzhenica	*Hemerocallis citrina*	*rps7*	0.032	0.0652	2.0375
Apostasia shenzhenica	*Asparagus officinalis*	*rps7*	0.032	0.0652	2.0375
Apostasia shenzhenica	*Allium cepa*	*rps12*	0.1699	0.0432	0.254267216
Apostasia shenzhenica	*Gastrodia elata*	*rps12*	0.0685	0.0321	0.468613139
Apostasia shenzhenica	*Hemerocallis citrina*	*rps12*	0.1685	0.04	0.237388724
Apostasia shenzhenica	*Asparagus officinalis*	*rps12*	0.1618	0.0804	0.496909765

Table S3. Prediction of RNA editing sites in *A.shenzhenica* mitogenome

Type	RNA -editing	Number	Percentage
hydrophobic	CTT (L) => TTT (F)	8	28.85%
	CCG (P) => CTG (L)	17	
	CCA (P) => CTA (L)	32	
	CCC (P) => TTC (F)	2	
	CCT (P) => CTT (L)	25	
	GCG (A) => GTG (V)	2	
	CCC (P) => CTC (L)	11	
	CTC (L) => TTC (F)	7	
	CCT (P) => TTT (F)	12	
	GCT (A) => GTT (V)	3	
	GCC (A) => GTC (V)	1	
hydrophilic	CGT (R) => TGT (C)	28	14.42%
	CGC (R) => TGC (C)	9	
	CAT (H) => TAT (Y)	15	
	CAC (H) => TAC (Y)	8	
hydrophobic-hydrophilic	CCC (P) => TCC (S)	13	10.82%
	CCA (P) => TCA (S)	8	
	CCT (P) => TCT (S)	16	
	CCG (P) => TCG (S)	8	
hydrophilic-hydrophobic	TCG (S) => TTG (L)	34	45.43%
	TCA (S) => TTA (L)	52	
	TCT (S) => TTT (F)	38	
	ACT (T) => ATT (I)	3	
	TCC (S) => TTC (F)	29	
	ACA (T) => ATA (I)	3	
	CGG (R) => TGG (W)	27	
	ACG (T) => ATG (M)	2	
	ACC (T) => ATC (I)	1	
hydrophilic-stop	CAA (Q) => TAA (X)	1	0.48%
	CGA (R) => TGA (X)	1	

Table S4 Distribution ofpenta and hexa SSRs in the *A.shenzhenica* mitogenome.

ID	SSR type	SSR	Start	End	Location
1	penta	(TCCGA)3	20000	20014	IGS(nad4L, nad5)
2	penta	(AAAAG)3	30671	30685	IGS(nad4L, nad5)
3	penta	(TCTTA)3	126755	126769	IGS(nad5, trnC-GCA)
4	penta	(TAAGG)3	191619	191633	IGS(nad9, tRNA-Thr)
5	hexa	(CCCCGG)3	225860	225877	IGS(trnF-GAA, rps7)
6	penta	(TAAAG)3	313314	313328	IGS(psaJ, rpl16)
7	hexa	(GGCTTG)3	324866	324883	IGS(rpl2, rps11)
8	penta	(TACAA)3	353917	353931	IGS(rps13, trnK-UUU)
9	penta	(TCATT)3	444355	444369	IGS(rps4, trnQ-UUG)
10	penta	(AAATG)3	543309	543323	IGS(rrn18, nad6)
11	penta	(TTTAG)3	579039	579053	IGS(ccmB, nad7)
12	penta	(GAAAA)3	582124	582138	IGS(ccmB, nad7)

Table S5 Distribution of perfect tandem repeats in *A.shenzhenica* mitogenome

**No**	**Repeat sequence**	**Size**	**Copy Number**	**Percent Matches**	**start-end**	**Location**
1	TATACATAGAAGCAA	15	2.7	100	173401--173441	IGS(nad9, tRNA-Thr）
2	ATATGTATTAG	11	2.4	100	174049--174074	IGS(nad9, tRNA-Thr）
3	ATAGATATAGAATAAGAATATATA	24	2	100	174101--174148	IGS(nad9, tRNA-Thr）
4	GAGGAGAAAGGAGGAATGCTCGACCGGAAGGGAACGAGTTACATACCTGGAACGAGTAGGTT	62	2.3	98	185579--185720	IGS(nad9, tRNA-Thr）
5	ATTTGACTATTTCA	14	1.9	100	203511--203537	IGS(nad9, tRNA-Thr）
6	TGTTACATTTTGT	13	1.9	100	204102--204126	IGS(nad9, tRNA-Thr）
7	ATAGTATATATAAA	14	2.1	100	268146--268174	IGS(matR, tRNA-Cys）
8	TCTTTCTGTCAA	12	2.1	100	305377--305401	IGS(atp4, trnW-CCA）
9	AATAGTTTTAATTATTATGTTGTTTC	26	2	100	418720--418771	IGS(trnM-CAU, rps4）
10	ATAATAATATTATTATTTTA	20	2.2	100	431112--431154	IGS(trnM-CAU, rps4）
11	TATAACTTTCTTATCCC	17	2.3	95	558125--558164	IGS(nad6, mttB）
12	CTACTAAAGAAGG	13	2.1	100	561059--561085	IGS(nad6, mttB）
13	TATATTGAATA	11	2.3	100	603499--603523	IGS(trnD-GUC, atp8）

Table S6 The NCBI accession numbers of mitogenomes used in this study

Famliy	Organism	Accession
Asparagus	*Allium cepa*	AP018390.1
	Hemerocallis citrina	MZ726801-3
	Gastrodia elata	MF070084-102
	Chlorophytum comosum	MW411187.1
	Asparagus officinalis	MT483944.1
Alismatales	*Zostera marina*	KX808392.1
	Stratiotes aloides	KX808393.1
	Spirodela polyrhiza	NC017840.1
	Butomus umbellatus	KC208619.1
Arecales	*Phoenix dactylifera*	MH176159.1
	Cocos nucifera	KX028885.1
Poales	*Zea perennis*	DQ645538.1
	Zea luxurians	DQ645537.1
	Tripsacum dactyloides	NC008362.1
	Zea mays	DQ645536.1
	Chrysopogon zizanioides	MN635785.1
	Coix lacryma-jobi var. puellarum	MT471098.1
	Sorghum bicolor	DQ984518.1
	Sporobolus alterniflorus	MT471321.1
	Eleusine indica	MF616338.1
	Oryza sativa	JN861112.1
	Oryza rufipogon	AP011076.1
	Oryza coarctata	MG429050.1
	Aegilops speltoides	AP013107.1
	Triticum aestivum	GU985444.1
	Hordeum vulgare	MN127974.1
	Lolium perenne	JX999996.1
	Bambusa oldhamii	EU365401.1
Outgroup	*Medinilla magnifica*	MT043351.1
	Eucalyptus grandis	NC040010.1
	Pyrus betulifolia	MW080658.1
	Nelumbo nucifera	NC030753.1
